# Estimating the burden of heat illness in England during the 2013 summer heatwave using syndromic surveillance

**DOI:** 10.1136/jech-2015-206079

**Published:** 2016-02-12

**Authors:** Sue Smith, Alex J Elliot, Shakoor Hajat, Angie Bone, Gillian E Smith, Sari Kovats

**Affiliations:** 1 Real-time Syndromic Surveillance Team, Public Health England, Birmingham, UK; 2 NIHR Health Protection Research Unit in Environmental Change and Health, Department of Social and Environmental Health Research, Faculty of Public Health and Policy, London School of Hygiene and Tropical Medicine, London, UK; 3 Extreme Events and Health Protection, Public Health England, London, UK

**Keywords:** SURVEILLANCE, MORBIDITY, EPIDEMIOLOGY

## Abstract

**Background:**

The burden of heat illness on health systems is not well described in the UK. Although the UK generally experiences mild summers, the frequency and intensity of hot weather is likely to increase due to climate change, particularly in Southern England. We investigated the impact of the moderate heatwave in 2013 on primary care and emergency department (ED) visits using syndromic surveillance data in England.

**Methods:**

General practitioner in hours (GPIH), GP out of hours (GPOOH) and ED syndromic surveillance systems were used to monitor the health impact of heat/sun stroke symptoms (heat illness). Data were stratified by age group and compared between heatwave and non-heatwave years. Incidence rate ratios were calculated for GPIH heat illness consultations.

**Results:**

GP consultations and ED attendances for heat illness increased during the heatwave period; GPIH consultations increased across all age groups, but the highest rates were in school children and those aged ≥75 years, with the latter persisting beyond the end of the heatwave. Extrapolating to the English population, we estimated that the number of GPIH consultations for heat illness during the whole summer (May to September) 2013 was 1166 (95% CI 1064 to 1268). This was double the rate observed during non-heatwave years.

**Conclusions:**

These findings support the monitoring of heat illness (symptoms of heat/sun stroke) as part of the Heatwave Plan for England, but also suggest that specifically monitoring heat illness in children, especially those of school age, would provide additional early warning of, and situation awareness during heatwaves.

## Introduction

Humans have efficient heat regulatory mechanisms which cope with increases in ambient temperature up to a particular threshold that varies by individuals depending on age, fitness and acclimatisation.^1^ The body increases radiant, convective and evaporative heat loss by vasodilatation and perspiration.^2^ The physiological and clinical effects of heat are generally well understood in healthy adults, but less so in children, persons with chronic or acute disease, and the elderly. As a result of the natural patterns of ageing (or senescence) on homeostatic mechanisms, normal thermoregulatory processes are compromised in elderly people, resulting in poorer heat tolerance.^3^ The age-related factors that contribute to this include poor aerobic fitness, differences in body composition and chronic health conditions. Children and babies have a limited ability to thermoregulate with children more at risk of dehydration than adults as they have a higher relative volume of water in their bodies.^2^ ^4^

### Heatwaves

Globally, heatwaves are responsible for impacting on human health on an annual basis: these impacts have been widely documented. During 1994, there were 3000 heat-related deaths in South Korea^5^; in 1995 there were in excess of 700 deaths in Chicago^6^; during 2003 a European heatwave resulted in 71 000 excess deaths including 15 000 in Paris;^7^ ^8^ more recently, a heatwave across India and Pakistan has resulted in excess of 3000 deaths, particularly in the elderly and poor.^9^ ^10^

The temperate maritime climate of the British Isles limits the probability of heatwaves occurring on the same scale as other continents, however, climate change models suggest that the probability of more severe heatwaves will increase in the future.^11^ Within the last few decades there have been a number of heatwaves that have occurred within England and Wales: the resulting impact on health has been documented in a number of studies.^12–17^ In particular, during the European heatwave of 2003, there was a 16% increase in excess deaths in England and Wales with London, and the elderly predominantly affected.^18^

In 2004, in direct response to the 2003 heatwave, and in particular the impact in France, the UK Department of Health launched the Heatwave Plan for England. The aim of the Plan is to support the National Health Service (NHS) and local authorities providing guidance and advice on how to prepare for and respond to heatwaves.^19^ An integral part of the Heatwave Plan for England is health surveillance: morbidity and mortality surveillance systems routinely monitor the health impact of heat during the summer.

### Syndromic surveillance

Syndromic surveillance is the near real-time collection, analysis, interpretation and dissemination of health-related data to enable the early identification of the impact (or absence of impact) of potential human or veterinary public-health threats which require effective public health action.^20^ Syndromic surveillance is becoming an important surveillance tool for monitoring public health in real-time. These systems have been previously used, albeit on a limited international basis, to monitor the health impact of heatwaves. In particular, work in France and the UK has used syndromic surveillance in real-time during heatwaves, with other studies assessing the potential of these systems to complement existing surveillance programmes.^21–27^

The objective of this paper was to describe the impact of the 2013 heatwave on morbidity utilising syndromic surveillance systems, using reported cases of heat illness presenting to healthcare services in England.

## Methods

### Syndromic surveillance systems

The Public Health England (PHE) Real-time Syndromic Surveillance Team (ReSST) coordinate a suite of national syndromic surveillance systems and deliver a real-time syndromic surveillance service that has been described in detail elsewhere.^28^ In brief, daily data are collected from a number of healthcare provider sources and analysed, interpreted and assessed using statistical algorithms (modelling historical data to identify significant activity).^29^ These data are aggregated into a number of syndromic indicators based on symptoms and clinical diagnosis of disease. For this study three national syndromic surveillance systems were used; general practitioner in hours (GPIH), GP out of hours (GPOOH) and emergency department syndromic surveillance systems (EDSSS) (table 1). The representativeness of the systems varied: GPIH had extensive coverage of the English population (monitoring approximately 65% of English patients) with good representativeness of regions across England; GPOOH had approximately 70% of GPOOH service providers across England albeit with less representative coverage across some English regions; the EDSSS was a sentinel surveillance system with 35 sentinel emergency departments (EDs) reporting across England and Northern Ireland, of which 18 were selected for use in this study (based on the suitability of the local clinical coding system being able to record symptoms of ‘heat illness’). Anonymised health data from each syndromic system were captured on a daily basis using automated processes: statistical models were applied to the data each day to identify significant exceedances compared to either recent activity, or historically expected levels.^30^ A baseline was estimated for each system and syndromic indicator using a multilevel hierarchical mixed effects model incorporating appropriate variables (eg, day of the week and public holidays).^30^ An upper 99% prediction interval threshold for expected activity each day was established using estimated baselines and variation in the daily data.^28^ ^29^

**Table 1:** PHE syndromic surveillance systems and associated reporting statistics

Syndromic surveillance system	Reporting statistic	Population coverage
GPIH	In hours (week days, daytime) GP consultation rates per 100 000 population	35 million (∼65% England pop)
GPOOH	Out of hours and unscheduled care (weekends, evenings/nights, public holidays) GP consultations for syndrome as a percentage of total consultations	∼70% coverage of GPOOH activity across England
EDSSS	Percentage of ED attendances coded to indicator.	35 EDs across England and Northern Ireland (18 used in this study)

EDSSS, emergency department syndromic surveillance system; GPIH, general practitioner in hours; GPOOH, general practitioner out of hours; PHE, Public Health England.

### Study period

A Heat-Health Watch alert system operates in England from 1 June to 15 September during which the UK Meteorological (Met) Office issues alerts indicating the likelihood of heatwave.^31^ There are four levels: level 1 (Heatwave and summer preparedness programme); level 2 (Heatwave is forecast—alert and readiness); level 3 (Heatwave action); and level 4 (Major incident—emergency response). Data for the June–September period in 2013 were compared with the same period in 2012 and 2014 (and additionally for 2011 for the GPOOH system only).

A heatwave occurred during July 2013 in England. Unusually hot weather affected most of the UK from 3 to 23 July 2013, making the month the third hottest July on record.^32^ Within the UK, the formal definition of a heatwave uses regional-specific criteria for a level 3 warning in the Heatwave Plan for England.^19^ Level 3 warnings were initially called on July 12–14. Maximum daily Central England Temperature (CET) exceeded 28.1°C from 13 to 19 July inclusive, and again on 22 July. During 18–19 July, the heatwave extended to Northern England, Wales, Scotland and Northern Ireland. A daily maximum temperature of 33.5°C was recorded on 22 July at Heathrow and Northolt (Greater London), which was the highest UK temperature recorded since 2006.^32^

### Syndromic data and data sources

For each of the three syndromic surveillance systems, daily counts of cases were obtained for heat/sunstroke (heat illness), an indicator that is based on an aggregation of underlying clinical codes extracted from the relevant system. Since several different clinical coding systems are used in general practice and in EDs the codes differ slightly between surveillance systems. A breakdown of the clinical codes underlying the syndromic indicator is available from the authors.

For the GPIH system the daily rate per 100 000 patient population was calculated. Accurate patient denominators are difficult to calculate for the EDSSS and GPOOH systems: it is difficult to define the ‘catchment population’ for individual hospitals/EDs, and individual OOH service providers can cover a number of different healthcare boundaries. Therefore, for each syndrome a daily percentage of all recorded contacts (attendances/consultations) was calculated.

Daily CET data (maximum °C) were obtained from the Met Office Hadley Centre observation data sets.^33^ The heatwave period was defined as 3–23 July 2013 for all England and Wales as this represented the period of highest temperatures. Not all regions were affected equally by the heatwave, and the North East was at level 1 throughout the period. However, limiting the analysis to just days with level 3 warnings would not have given sufficient power for the analysis.

### Trend analyses

Time series graphs showing daily data for heat illness and the 7-day moving averages of rates/percentages were plotted over the study period during the years 2012–2014 for the EDSSS, GPOOH and GPIH surveillance systems. Trends were initially visually examined to identify whether heat illness parameters had increased during the 2013 heatwave, and further compared to other years to identify inter-annual differences. Trends were also compared with daily temperatures (CET). GPIH syndromic surveillance data were further analysed by age (age groups 0–4, 5–14, 15–64, 65–74, and ≥75 years).

### Estimation of burden of heat illness

To estimate the total burden of heat illness, the total number of cases in the GPIH system was calculated from 1 June to 15 September period for each of the years 2012–2014. The number of GP practices contributing to the GPIH increased over these years so the percentage coverage of England was estimated for this summer period each year using the mean weekly GPIH population for weeks 23–37 (broadly corresponding to 1 June through 15 September) as the numerator and national estimates of patient registration (England) as the denominator. The calculated percentage coverage was used to estimate the total heat illness cases for England over that period, assuming that all areas of the country were equally affected by hot weather, and that the rest of the country had equivalent GP consultation rates. This estimate was not undertaken for the EDSSS and GPOOH systems due to the difficulties in estimating accurate population denominators and the sentinel nature of the EDSSS.

### Incidence rate ratios

Incidence rate ratios (IRR) were calculated for the GPIH system using the mean weekly incidence rate (per 100 000 population) for heat illness for June-September (the Heat-Health Watch period) during the year of the heatwave 2013. This was repeated for the same period in the non-heatwave years 2012 and 2014. An IRR was calculated by comparing the mean values for the heatwave and non-heatwave years and was stratified by age group. The Taylor method was used for calculating 95% CIs for the IRR,^34^ with the assumption that there was independence between years so that the covariance term was zero.

## Results

### Heat illness during the 2013 heatwave

**Figure 1:**
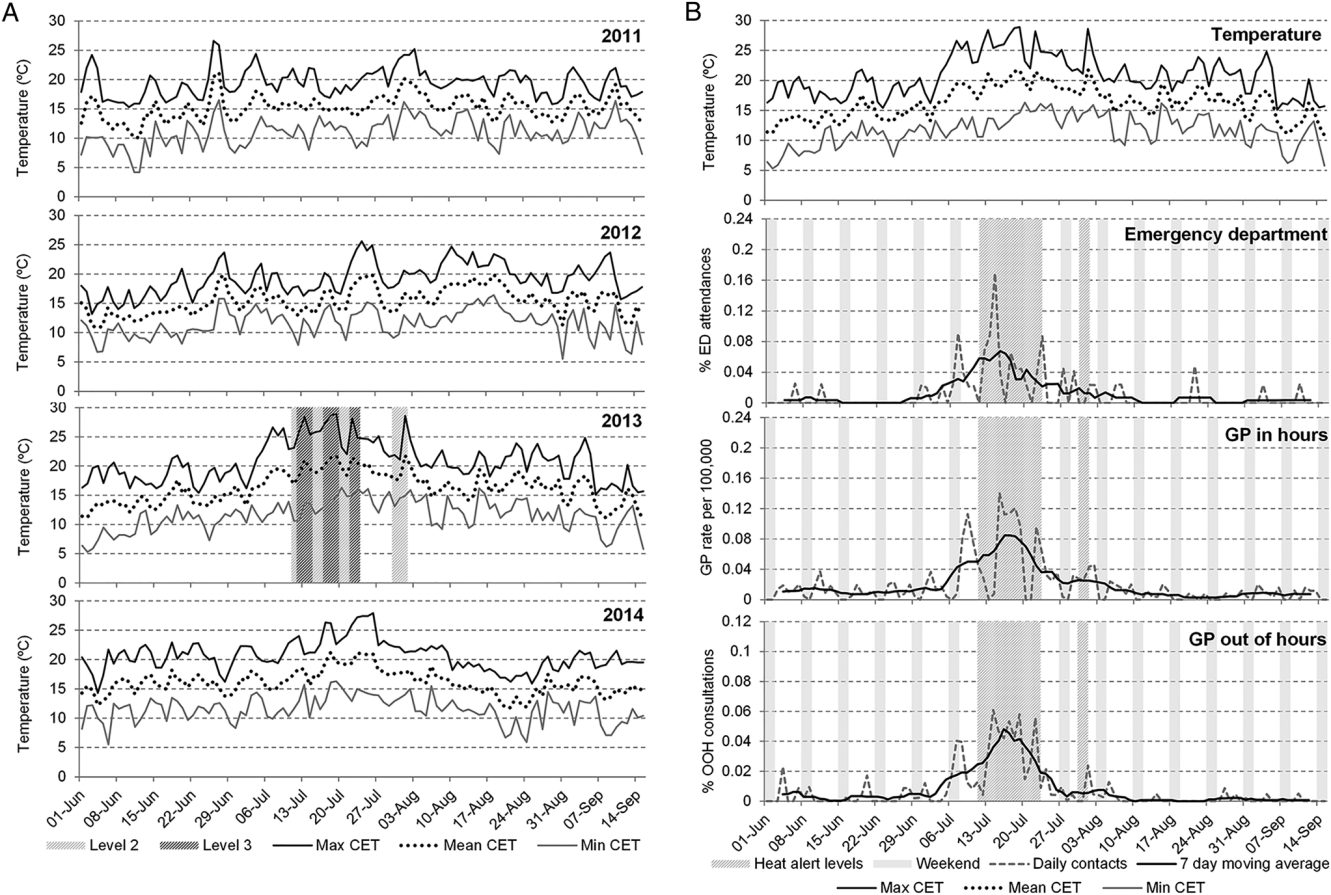
Daily Central England Temperature (maximum, mean and minimum) for summers 2011–2014 and Heat-Health Watch alert levels for summer 2013 (A). Daily incidence and 7-day moving average of heat illness (all ages) for 2013. Heat alert periods (heat health alert levels 2/3) for 2013 are indicated by hashed grey bars; weekends are indicated by solid grey bars (B). Daily Central England Temperature (maximum, mean and minimum) for 2013 are illustrated in the top panel.

All three syndromic surveillance systems (GPIH, GPOOH and EDSSS) recorded daily peaks in heat illness which coincided with highest daily temperatures during summer 2013. Temperatures started increasing on 3 July, with the first peak in temperature on Sunday 7 July (26.6°C maximum daily CET; figure 1A): EDSSS and GPOOH heat illness indicators peaked concurrently on Sunday 7 July (figure 1B), and GPIH on Tuesday 9 July. The level 3 heatwave alerts were called on 12–14 July. The EDSSS and GPOOH systems recorded further peaks in heat illness activity on Sunday 14 July. GPIH recorded a second peak in heat illness consultations on Monday 15 July (as GP consultations do not routinely take place at weekends), and were high for the remainder of that week. A further peak in GPIH on Thursday 18 July (coincided with a second level 3 alert period from 17–19 July).

A final level 3 alert was issued from 22 to 23 July which coincided with peaks in GPIH and GPOOH (22 July) and ED (23 July). The GPOOH recorded a small spike in heat illness 1 August when CET reached 28.6°C, and GPIH consultations for heat illness also showed an increase over 1–2 August.

### Heat illness consultations by age group

**Figure 2:**
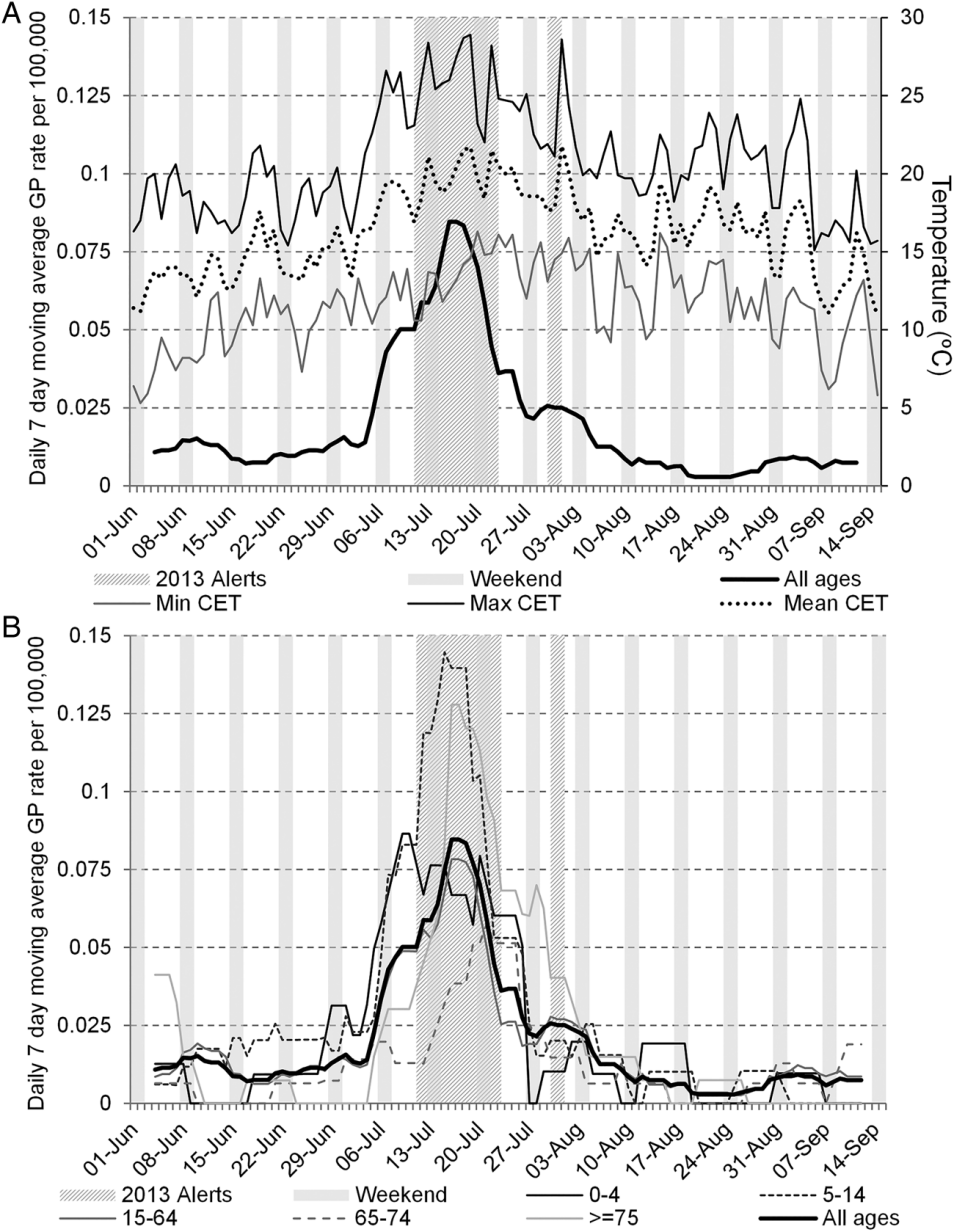
GP in hours daily heat illness consultations (7-day moving average): (A) all ages (with Daily Central England Temperature illustrated); and (B) by age group during the 2013 Heat-Health Watch period (1 June–15 September 2013). Heat alert periods (heat health alert levels 2/3) for 2013 are indicated by hashed grey bars; weekends are indicated by solid grey bars. GP, general practitioner

Cases of heat illness in the GPIH increased across all age groups during the heatwave period, but the highest rates were in school children (5–14 years) and those aged ≥75 years (figure 2). Children showed more sensitivity to increases in temperature with rates in the 0–4 and 5–14 years age groups rising sharply in the early phase of the heatwave from the first temperature peak on 7 July.

GPIH consultation rates for heat illness reached a peak in the 0–4 years age group on 9 July while the peak in the 5–14 years age group occurred at a lag of 6 days, on 15 July. Cases in adults rose more slowly with the peak occurring a day later on 16 July for the 15–64 and ≥75 years age groups while the 64–75 peaked towards the end of the heatwave period on 21 July. While other age groups saw rates of heat illness return to normal after the final level 3 alert on 23 July, rates in ≥75 years remained higher than other ages up to the beginning of August.

### Estimating the burden of heat illness in England

The number of GPIH consultations for heat illness were estimated for each year 2012–2014 (table 2). Unsurprisingly, the burden was greatest in the hottest year (2013), with nearly twice the number of consultations compared to non-heatwave years.

**Table 2:** Estimated number of GPIH heat illness consultations during summer (1 June to 15 September) 2012–2014

GPIH percentage population coverage of England*†			Actual consultations			Estimated total population consultations (95% CIs)		
2012	2013	2014	2012	2013	2014	2012	2013	2014
39	43	53	135	500	326	345 (287 to 403)	1166 (1064 to 1268)	616 (549 to 683)

*Estimated coverage based on average GPIH weekly population weeks 23–37 and numbers of patients registered at GP practices in England in July for years 2012–2014.

†Source of England population data: Health and Social Care Information Centre.

GPIH, general practitioner in hours.

Based on population figures for England, there were an estimated total of 1166 (95% CI 1064 to 1268) GP consultations for heat illness during summer 2013. The estimated figure for 2014 and 2012, respectively, were: 616 (95% CI 549 to 683) and 345 (95% CI 287 to 403). These estimates assume that all regions of England were equally affected by hot weather during these 3 years.

### Incidence rate ratios

Incidence rate ratios (IRR) for the GPIH showed 2.5 times as many heat illness cases in 2013 than in non-heatwave years (table 3). The greatest difference was in the elderly, with 3.2 times as many cases in those ≥75 years, and in the young where the 0–4 years age group had three times as many cases in the heatwave year (however, please note that all IRR results were statistically non-significant).

**Table 3:** Mean incidence of heat illness in 2013 by syndromic surveillance system by age group and IRR for heat illness in GPIH (comparing heatwave (2013) and non-heatwave (2011, 2012 and 2014) years)

	GPOOH	EDSSS	GPIH	
Age group	Mean consultations (% of total)	Mean attendances (% of total)	Mean incidence rate (per 100 000)	IRR (CI 95%)
0–4	0.02	0.05	0.14	3.0 (−7.5 to 13.5)
5–14	0.08	0.06	0.19	2.6 (−5.4 to 10.6)
15–64	0.03	0.05	0.13	2.3 (−3.1 to 7.7)
65–74	0.02	0.1	0.09	2.4 (−3.7 to 8.4)
75 plus	0.01	0.05	0.16	3.2 (−7.0 to 13.5)
All ages	0.03	0.05	0.14	2.5 (−3.4 to 8.4)

EDSSS, emergency department syndromic surveillance systems; GPIH, general practitioner in hours; GPOOH, general practitioner out of hours; IRR, incidence rate ratios.

## Discussion

Despite the projected increase in the frequency of heatwaves, and the occurrence of several devastating heatwaves over the last few decades, there is still a knowledge gap in the impact of heatwaves on morbidity.^35^ This paper contributes to the evidence showing that heat illness has a small burden in England during average summers, but this increased during hot summers (to approximately 1166 GP consultations over the summer of 2013). These findings support the need for public health advice (the Heatwave Plan for England)^19^ and to ensure that GPs can identify and treat the condition to allow early intervention. These estimates could also be usefully used to inform GPs and ED clinicians of predicted numbers of patients requiring treatment for heat illness symptoms during heatwave periods. The results also revealed increasing rates of heat illness prior to a level 3 alert, supporting required vigilance during level 2 alerts (‘Alert and Readiness’), at which there will be increasing presentation of heat illness in the community, which will continue and increase into the level 3 alert period.

The age profile of the heat illness consultations was similar to what is known from physiological studies, and also mortality risks.^36^ The high rates in school age children requires some further investigation: this supports the advice provided in the Heatwave Plan for England that younger and school-age children are particularly at risk from heat, however, it might suggest that additional information and advice might be needed for those providing support and services for children. The 2013 heatwave occurred when children were at school. It is not clear whether this would have impacted on the results, however, it is possible that poorly ventilated school buildings, wearing school uniform and participating in school-based physical activity programmes might have accentuated any heat illness particularly in children aged 5–14 years. For future work, it would be important to compare the impact of heatwaves occurring during term-time and holiday periods to ascertain when there is greater risk of heat illness to school children. This information could further influence the advice provided in heat plans specifically for the protection of school-aged children.

The differential timing of the presentation of age groups during the heatwave also provided interesting discussion. In general, parents are more likely to present their children earlier to healthcare services, while the elderly are more likely to delay presentation to avoid burdening services.^37^ This might have influenced the presentation of heat illness cases in this study, however, this also merits further investigation.

There are some limitations to this work. First, the validity of the heat illness indicator: it is unlikely to be true heatstroke (where the core body temperature reaches 40°), but more likely heat exhaustion or classical heat illness. Further research could investigate what actual conditions are being represented, and possible comorbidities. There is also some uncertainty about the national estimates for GP consultations, which are extrapolated from sentinel sites around England: it is also incorrect to assume a similar impact of heat across all regions in England, some regions did not experience level 3 alerts. For the calculation of IRRs we used a comparison of the summer periods, rather than specific heatwave periods. This was due to the fact that limiting the analysis to the latter period would have reduced the numbers of cases available for analysis, however, non-heatwave summer weeks may have had a masking effect on the results. Finally, the incidence of heat illness cases recorded within the population is relatively infrequent resulting in small numbers of cases with resulting analyses often resulting in wide CIs.

Syndromic surveillance can provide intelligence on the impact of the heat that is, prolonged periods of heat, or higher minimum temperatures may have a greater impact than a short but hotter spell. GPOOH consultations and ED attendances for heat illness were timely with respect to the increase in temperatures, but GPIH consultations reacted more slowly. This is consistent with expected healthcare seeking behaviour, and provision of health services, with more severely ill patients preferentially visiting EDs and unscheduled GP services (particularly during weekends) rather than booking next available routine GPIH appointments. These results strengthen heatwave planning and the attention required on different syndromic surveillance systems depending on the timing of a heatwave period.

Our results indicate that all ages should be monitored during a heat alert, but there is a particular requirement to monitor the elderly once the heat alert (level 3) has finished, due to the lag in morbidity presenting after the heat alert has passed. This prolonged lag differs from the short lag shown between temperature and mortality, which often peaks on the same day, or one day later.^38^ In addition, it is apparent from these results that the health impact of heatwaves can be seen prior to the notification of heatwave conditions that is, a level 3 alert, and therefore the advice to health service providers should include taking preventative measures before these levels are reached. Our results also indicate that heat illness in children aged 0–4 and 5–14 years could be a better indicator to provide early warning of increases in heat burden in the general population. These results may be useful in future evaluations of the Heatwave Plan for England and also for the many countries that have now adopted heatwave plans and early warning systems.^39^

**What is known on this subject**

- Heatwaves can impact on human health causing increases in morbidity and mortality. Although there is strong evidence linking extreme heat with excesses in mortality, there is less literature describing the impact on morbidity, including the impacts on specific age groups and demand on specific health care services during heatwaves.

**What this study adds**

- The presentation of heat illness symptoms to healthcare services was highest in children and the elderly. General practitioner out of hours consultations and sentinel emergency department attendances for heat illness were timely with respect to the increase in temperatures, but GP in hours consultations exhibited a lag. The health impact of heat increased shortly before the level 3 alert and, in the elderly population, persisted after the conclusion of heatwave conditions suggesting that further care of this population is required following the cessation of heat alerts.
